# Coexistence of Reward and Unsupervised Learning During the Operant Conditioning of Neural Firing Rates

**DOI:** 10.1371/journal.pone.0087123

**Published:** 2014-01-27

**Authors:** Robert R. Kerr, David B. Grayden, Doreen A. Thomas, Matthieu Gilson, Anthony N. Burkitt

**Affiliations:** 1 NeuroEngineering Laboratory, Department of Electrical and Electronic Engineering, University of Melbourne, Melbourne, Australia; 2 Centre for Neural Engineering, University of Melbourne, Melbourne, Australia; 3 NICTA, Victoria Research Lab, University of Melbourne, Melbourne, Australia; 4 Bionics Institute, Melbourne, Australia; 5 Department of Mechanical Engineering, University of Melbourne, Melbourne, Australia; 6 Laboratory for Neural Circuit Theory, RIKEN Brain Science Institute, Saitama, Japan; Georgia State University, United States of America

## Abstract

A fundamental goal of neuroscience is to understand how cognitive processes, such as operant conditioning, are performed by the brain. Typical and well studied examples of operant conditioning, in which the firing rates of individual cortical neurons in monkeys are increased using rewards, provide an opportunity for insight into this. Studies of reward-modulated spike-timing-dependent plasticity (RSTDP), and of other models such as R-max, have reproduced this learning behavior, but they have assumed that no unsupervised learning is present (i.e., no learning occurs without, or independent of, rewards). We show that these models cannot elicit firing rate reinforcement while exhibiting both reward learning and ongoing, stable unsupervised learning. To fix this issue, we propose a new RSTDP model of synaptic plasticity based upon the observed effects that dopamine has on long-term potentiation and depression (LTP and LTD). We show, both analytically and through simulations, that our new model can exhibit unsupervised learning and lead to firing rate reinforcement. This requires that the strengthening of LTP by the reward signal is greater than the strengthening of LTD and that the reinforced neuron exhibits irregular firing. We show the robustness of our findings to spike-timing correlations, to the synaptic weight dependence that is assumed, and to changes in the mean reward. We also consider our model in the differential reinforcement of two nearby neurons. Our model aligns more strongly with experimental studies than previous models and makes testable predictions for future experiments.

## Introduction

Operant conditioning refers to an individual modifying its behavior based on some consequence of that behavior. Understanding how this process arises from neural mechanisms in the brain will provide a promising step toward linking neural mechanisms with behavior and learning and discovering how the brain gives rise to cognitive functions in general. It is also applicable to brain-computer interfaces, where operant conditioning can be used to develop control of external prostheses rather than tailoring them to existing neuronal circuitry [1].

Operant conditioning experiments have shown that the firing rate of individual neurons in the precentral motor cortex and prefrontal cortex of monkeys could be significantly increased by giving positive reinforcement, provided that the monkeys were also given immediate feedback on the neuron's firing [2]–[4]. A visual display presented the monkeys with a time-decaying signal that was incremented for each action potential that an implanted electrode measured. Upon reaching a threshold value, the signal was reset and the monkey received a food reward. Negative punishment (i.e., the removal of reward in order to decrease a particular behavior) was performed with a similar setup, where measured spikes decremented the signal (and artificially generated spikes incremented the signal) [3]. In this case, low firing rates were elicited. Through a combination of positive reinforcement and negative punishment, they also showed that a differential between the firing rates of two neurons could be elicited.

Current theories hold that learning at the behavioral level is ultimately due to changes at the synaptic level. Reinforcement learning models of synaptic plasticity depend on neuronal activity and also on a reward signal [5] that, due to the evidence linking dopamine to reward learning in the brain [6], typically represents the amount of extracellular dopamine present. Similar to Fremaux et al. [7], we identify two main types of existing models. First, there are models that have been derived theoretically to maximize the received reward [8]–[11], such as the R-max model [7]. Secondly, there is reward-modulated spike-timing-dependent plasticity (STDP) [11]–[13], or RSTDP, where the amplitudes of synaptic changes that would have been made by STDP [14], [15] are modulated by subsequent rewards.

A reinforcement learning model of synaptic plasticity exhibits unsupervised learning (i.e. learning that occurs independently of any rewards) if there is long-term potentiation (LTP) or long-term depression (LTD) at the mean reward level. Additionally, for models where LTP and LTD do not depend on the current synaptic weight (additive models), unsupervised learning is only present if the LTP and LTD do not cancel with each other. Studies with existing models find that there should be no unsupervised learning in order to perform reinforcement learning tasks, such as the operant conditioning of neuronal firing rates [7], [16]. However, even after development, the brain receives large amounts of novel sensory information without any associated rewards or punishments [17]. Any learning based on this information is necessarily unsupervised, suggesting an ongoing role for unsupervised learning after development. This likely depends on the brain region. In synapses onto GABAergic spiny neurons in the rat striatum, Pawlak and Kerr [18] showed that no LTP or LTD occurred when D1-receptors (dopamine receptors) were blocked. In synapses onto pyramidal neurons in the rat hippocampus, however, Zhang et al. [19] observed classical STDP learning windows without any dopamine present. When extracellular dopamine was added, Zhang et al. [19] observed increased LTP for pre-post spike pairs and that LTD had switched to LTP for post-pre spike pairs. Based on this, it seems unlikely that there would be no LTP or LTD at the base level of dopamine, which suggests that unsupervised learning can coexist with reward learning.

Here, we consider the case where unsupervised learning does occur (unlike in the situation considered in previous studies [7], [16]) and so, even without reinforcement learning, a balance of LTP and LTD produces stable firing rates. Under this assumption, we demonstrate that existing RSTDP models are unable to elicit increased firing rates in neurons that are rewarded for firing. We propose a new RSTDP model that can elicit reinforcement learning, in which LTP and LTD are modulated separately by the reward signal. This is more consistent with the experimental observations that dopamine affects LTP and LTD differently, even causing LTD to switch to LTP for high concentrations [19]. We show that these findings are robust to the introduction of spike-timing correlations, the synaptic weight dependence that is assumed, and the reward signal used. We demonstrate that our model is also able to reproduce the differential reinforcement of two neurons observed by Fetz and Baker [3]. Finally, we compare the learning induced by the operant conditioning of firing rates using our model with the R-max model to highlight the impact of including unsupervised learning with reward learning.

## Results

### RSTDP Model

**Figure 1:**
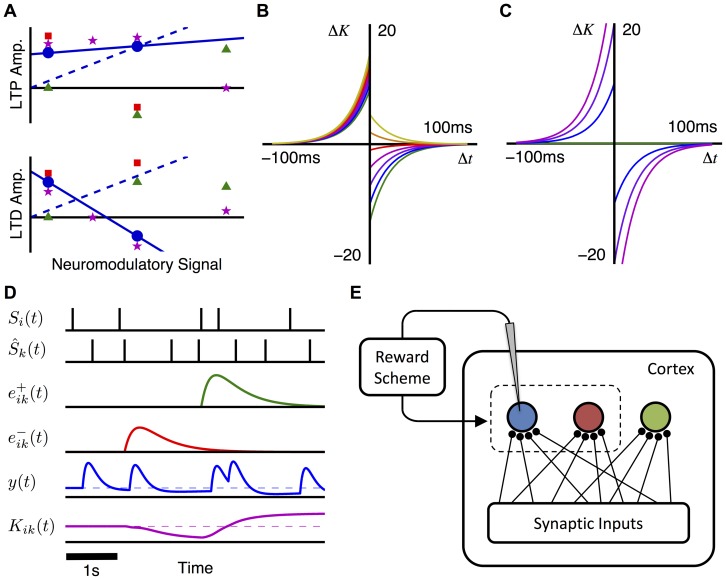
Modulation of STDP. **A:** Qualitative summary of the observed modulation of LTP and LTD amplitudes with increasing concentrations of dopamine (blue circles), octopamine (red squares), norepinephrine (green triangles), and acetycholine (magenta stars). These are based on observations by Zhang et al. [19], Cassenaer and Laurent [42], Salgado et al. [43], and Sugisaki et al. [41], respectively. The markers show qualitative effects only and the scales between the different modulators are not necessarily comparable. An example of our new RSTDP model parameterized to exhibit the same effect on STDP as dopamine (solid blue line). This is compared to an example of classical RSTDP model (dashed blue line). **B:** Effective learning windows for dopamine RSTDP for reward levels of 0 (green), 1 (blue), 2 (purple), 3 (magenta), 4 (red), 5 (orange), and 6 (yellow). The modulation factors are 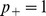, 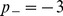, 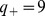, and 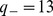. **C:** Effective learning windows for classical RSTDP. Same axes and lines (not all are shown) as in B. The modulation parameters are 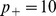, 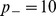, 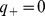, and 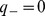. **D:** Conceptual plot of RSTDP variables during an operant conditioning experiment. Variables are (from the top down): post- and pre-synaptic spike trains, LTP and LTD eligibility traces, reward signal (dashed line shows the mean value), and synaptic weight (dashed line shows the initial value). **E:** Feedforward network where reinforced neuron (blue) is recorded from, determining the reward, which in turn influences changes made to the synapses into the reinforced and surround (red) neurons. The control neuron (green) represents either neuron before the operant conditioning experiment was preformed.

To better incorporate the effects that neuromodulators have been observed to have on synaptic plasticity (Figure 1A), we propose a new RSTDP model in which LTP and LTD can be modulated differently by a neuromodulator (e.g., dopamine). In this model, there are a pair of modulated parameters for each of LTP and LTD. Each pair describes the linear effect that a neuromodulator has on the amplitude of LTP and LTD. The modulation offsets, 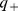 and 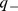, give the amplitudes of LTP and LTD, respectively, when the reward signal is zero. The modulation slopes, 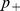 and 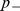, give the rates of change of the amplitudes of LTP and LTD, respectively. By setting both modulation offsets to zero (i.e., 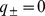), the classical RSTDP model is recovered (dashed blue line in Figure 1A). In this paper, we focus on a particular set of modulation parameters (solid blue line in Figure 1A) that leads to the effect that Zhang et al. observed dopamine to have on STDP [19] (blue circles in Figure 1A). We refer to this parameterization as dopamine RSTDP. Figure 1B illustrates the effective learning windows corresponding to changes in the reward signal, as compared to classical RSTDP shown in Figure 1C.

**Figure 2:**
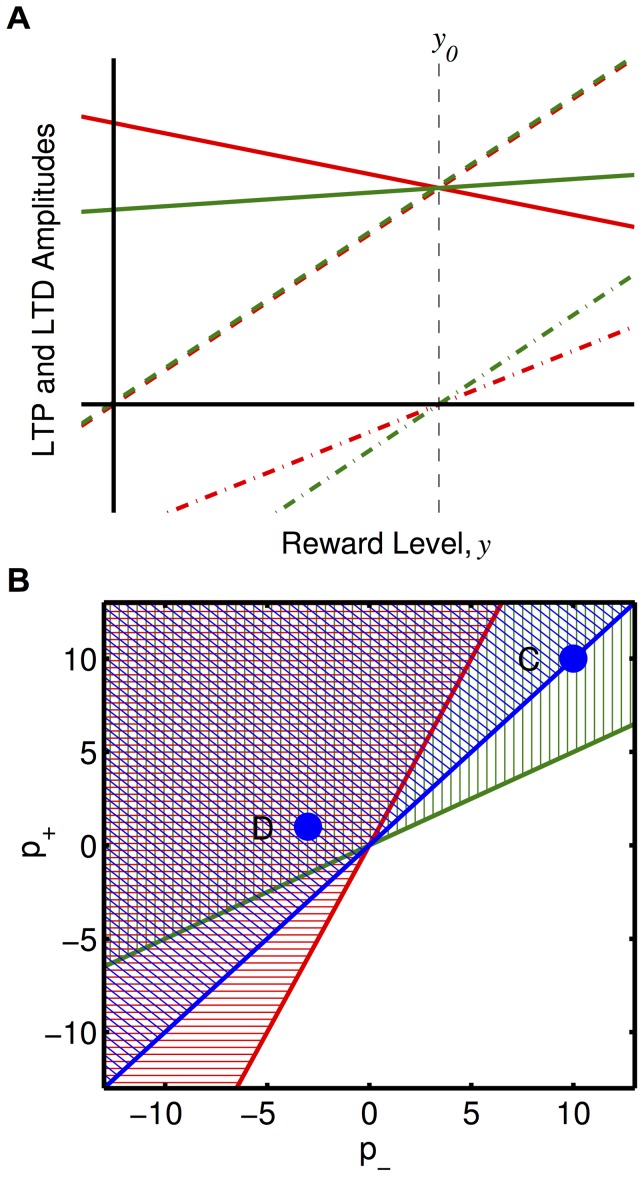
Comparison of our RSTDP model and classical RSTDP models. **A:** Amounts of LTP (green) and LTD (red) vs. reward level, with our RSTDP model (solid) and with classical RSTDP with and without unsupervised learning (dashed and dot-dashed, respectively) at the equilibrium synaptic weight. For classical RSTDP without unsupervised learning the reward signal has been shifted such that there is no LTP and LTD at the base reward level, 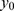 (vertical, black, dashed line) instead of at zero reward, 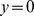. **B:** An increase (decrease) in firing rate is predicted to occur in the hatched (unhatched) regions for LTP:LTD ratios at the base reward level (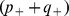: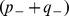) of 2:1 (red), 1:2 (green), and 1:1 (blue). On the lines that divide these regions no increase or decrease is predicted. The points marked as C and D correspond to a base level ratio of 1:1 and represent the classical and dopamine parameter sets used in this paper.

Our new RSTDP model introduces two qualitatively new features. The first is that there can be LTD and LTP when the reward is zero (provided that 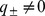). This differs from previous studies in which firing rate reinforcement was demonstrated [7], [16], where the base reward signal was zero and, at this level, there was no LTP or LTD. This difference is illustrated in Figure 1A and Figure 2A. However, we consider the case where the base reward level is positive and so, for both our RSTDP model and classical RSTDP, there is LTD and LTP present at the base reward level and, therefore, there is unsupervised learning. The second new feature, introduced by our new RSTDP model, is that LTD and LTP are modulated separately by the reward signal. This means that it is possible for a balance of LTP and LTD to be disrupted by an increase (or decrease) in reward. It also means it is possible for the LTP (LTD) caused by pre-post (post-pre) spike pairs to be differentially switched to LTD (LTP) for high reward signal values. The latter of these, where LTD transitions to LTP, is demonstrated with dopamine RSTDP (Figure 1B) and matches observed effects of dopamine of STDP [19]. In classical RSTDP, the only point at which both LTP and LTD switch is when the rewards become negative (or below baseline in previous studies [7], [16]).

The model is able to exhibit differential modulation of LTP and LTD because it stores the effects of the pre-post and post-pre spike pairs in two separate eligibility traces, 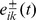. This is in contrast to classical RSTDP, which combines these effects into a single eligibility trace. Figure 1D shows the two eligibility traces for an individual synapse, as well as the reward signal, 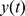 (determined by the post-synaptic spike train, 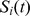), and the changes elicited in the synaptic weight, 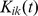.

### Analytical Predictions

To apply this model to operant conditioning experiments, we considered the feed-forward network shown in Figure 1E, containing three different types of post-synaptic neurons:

- **Reinforced:** The firing of the reinforced neuron is recorded and determines the amount of reward delivered. In operant conditioning experiments, the firing rate of this neuron was observed to increase.
- **Surround:** The surround neuron is located near the reinforced neuron but its firing does not affect the reward delivered.
- **Control:** The control neuron represents either the reinforced or surround neuron before the operant conditioning experiment was performed.

Each spike from the reinforced neuron produced a perturbation of the reward signal, referred to as the reward kernel. The reward kernel has a mass, 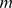, between 0 and 1. We initially focussed on the case where 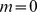 and hence the mean of the reward signal, 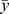, is equal to the base level, 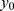. This is the case in Figure 1D, where the kernel has a negative tail. The kernel is scaled by a reward strength, 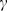, which is positive to reinforce a high firing rate and negative to reinforce a low firing rate.

Analytically, we found that, for there to be reinforcement and unsupervised learning, rewards must produce a large increase in LTP then LTD and the reinforced neuron's firing must be irregular. We determined this by considering the changes to the mean feed-forward weight into neuron 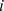, which is given by 
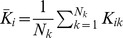
, where there are 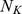 inputs and 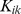 is the weight from input 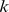 to neuron 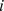. Focussing on the case where the inputs to the neurons are uncorrelated, this mean weight evolves according to (see Section 1 of Text S1 for derivation) 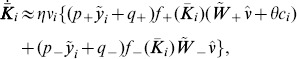
(1)


where 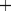 and 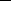 refer to the LTP and LTD parts of the learning window, respectively, 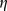 is the learning rate, 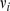 is the firing rate of neuron 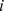, 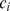 is the normalized, mean strength of the cross-covariances between neuron 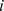 and its inputs, 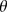 describes the effect of these cross-covariances on learning, 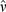 is the input firing rate, 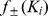 and 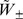 are the weight dependence function and mass of the learning window parts, respectively, and 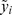 gives the mean effective reward following the spikes of neuron 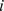. For weights into the control and surround neurons, 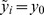 and 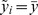, respectively. For weights into the reinforced neuron, 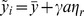, where 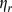 describes the interaction between the reward kernel and the eligibility kernel, and 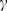 and 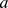 are the reward strength and the net area of the auto-covariance function of the reinforced neuron, respectively. The statistic 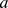 provides a measure of irregularity in the firing of a neuron. In this way, reinforcement of a neuron occurs based on the average value of the reward signal following spike pairs.

We consider the case where the mean firing rates of the inputs are equal and only small spike correlations exist. In this case, the firing rate of a neuron is dependent on the mean excitatory synaptic weight of its inputs (assuming no, or fixed, inhibitory inputs). Therefore, for the reinforced neuron to increase its firing rate for a given set of inputs, the mean weight into it must increase compared to the mean weight into the control neuron. From Equation (1), this requires that 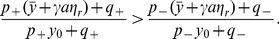
(2)


Assuming that 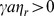 and that 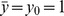, the requirement for reinforcement given by Equation (2) can be further simplified as 
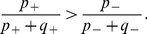
(3)


In classical RSTDP, where 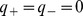, this requirement cannot be satisfied and neither an increase nor a decrease in the reinforced firing rate will occur. This is because, in classical RSTDP, LTP and LTD must both be zero at the same reward level and so, for there to be linear modulation of LTP and LTD that produces a balance of LTP and LTD at the base reward level, LTP and LTD necessarily match/balance at any reward level (dashed lines in Figure 2A). In the study by Legenstein et al., the reward signal was shifted so that there was zero LTP and LTD at the base level (dot-dashed lines in Figure 2A) and so, except at this point, no balancing of the amounts of LTP and LTD were necessary [16]. In that case, reward above the base level produced Hebbian STDP while reward below the base level produced anti-Hebbian STDP. Therefore, provided that correlations between the inputs and the neurons caused there to be a greater amount of LTP than LTD while the reward was above the base level, RSTDP would lead to a stable increase in the synaptic weights and the firing rate of the reinforced neuron. However, in this situation, no unsupervised learning was present, as there was no LTP and LTD at the average reward level. If, in the study by Legenstein et al. [16], the reward signal had not been shifted and there was LTP and LTD at the base reward level, unsupervised learning would be present but there would not be a balance of LTP and LTD at the base reward level. In this situation, the synaptic weights would either grow or decay unstably even without any rewards being given to the system.

In our RSTDP model, LTP and LTD are not necessarily both zero at the same reward level and so, to balance each other at the base reward level, they are not required to balance for all reward levels (solid lines in Figure 2A). In this case, it depends on the particular parameters as to whether reinforcement occurs or whether the ‘rewards’ actually behave as punishments and lead to a decrease in the firing rate of the neuron. For the dopamine inspired modulation parameters that we focus on, this requirement is met and reinforcement occurs. The inequality in Equation (3) and the illustration in Figure 2B show that, relative to the amounts of LTP and LTD at the base reward level, the increase in the amount of LTP with reward must be greater than the increase in the amount of LTD in order for the firing rate to increase (be reinforced). If the increase in LTP is the same as (less than) the increase in LTD, then the firing rate remains the same (decreases). Therefore, the parameters we consider here, which correspond to the results of Zhang et al. [19], are just one of many possible sets of modulation parameters that we predict would lead to firing rate reinforcement.

Figure 1B shows that, for high values of dopamine, there is only LTP (post-pre spike pairs lead to LTP, instead of LTD). Because of this, if 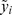, the mean effective reward following the spikes of neuron 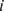, is sufficiently large then on average post-pre spike pairs with neuron 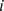 would lead to LTP and weights into neuron 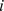 would grow in an unstable manner. However, we found that there is a broad range of modulation parameters for which a stable fixed point for the mean input weight exists.

In addition to the modulation parameters, Equations (1) and (2) predict that the amount of reinforcement that occurs depends on the value of 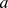, which we show depends on how irregular the firing of the reinforced neuron is.

### Operant Conditioning Simulations

**Figure 3:**
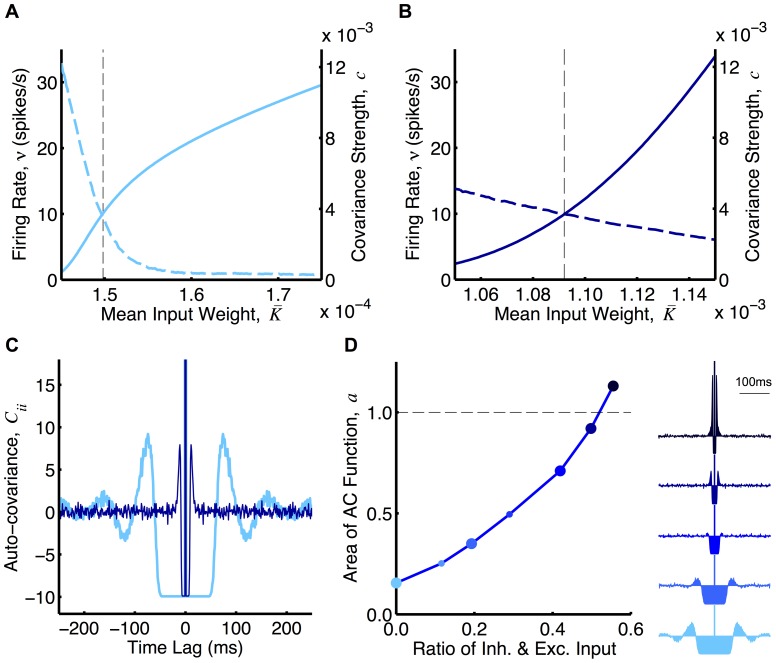
Numerically determined spiking statistics using the LIF neuron model. **A:** Mean output firing rate (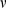, solid) and mean cross-covariance strength (covariance normalized by the firing rate) between the input and output spike trains (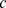, dashed) for different mean input weights, 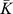, for a LIF neuron with 10,000 excitatory inputs. **B:** Same as A but for a LIF neuron with 8,000 excitatory inputs and 2,000 inhibitory inputs. **C:** The auto-covariance function of the output spike trains, 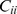, of the LIF neurons in A (light blue) and B (dark blue) with mean input weights of 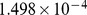 and 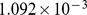, respectively (dashed vertical lines in A and B). **D:** The net area of the auto-covariance (AC) functions, 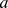, of LIF neurons (with input and output rates of 10 spikes/s) with 8,000 excitatory inputs and 2,000 inhibitory inputs for different ratios of the inhibitory and excitatory input currents. The auto-covariance functions for the first, third, fifth, sixth, and seventh points are shown to the right from bottom to top. The first point is the case in A and C (light blue), except with only 8,000 excitatory inputs, and the fifth point is the case in B and C (dark blue). Table 1 shows the parameters used in the LIF neuron model.

To support our analytical predictions, we simulated the learning during the operant conditioning of a neuron's firing rate using leaky integrate-and-fire (LIF) neurons in two different cases. In the first, the neurons received 10,000 excitatory inputs (E), while in the second, they received 8,000 excitatory and 2,000 inhibitory inputs (E+I). In the E+I case, only the excitatory input weights changed due to RSTDP (i.e., the inhibitory inputs' weights were fixed). While we assume no covariance between the inputs, the correlations arising due to the influence of individual input spikes on the firing of the output neuron (spike triggered correlations) are significant and need to be taken into account. Figures 3A and 3B show numerically determined values for the strengths of these correlations (normalized by the firing rate) varying with mean input weight for the two cases. While the correlation strength increases with the mean input weight, it does so in a weaker fashion than the firing rate and so the normalized correlation strength decreases with mean input weight. The auto-covariance functions of the LIF neurons had a negative region for short time lags (Figure 3C). Negative regions represent spike time differences that are less likely to occur. In the integrator regime (E), the negative region is due to a minimum inter-spike-interval exhibited by the neuron. This minimum inter-spike-interval was smaller in the E+I case than the E case because the neuron exhibited more irregular firing. The net area of the auto-covariance function, 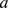, is affected by the irregularity in firing: lower values occur for more regular firing and higher values for more irregular firing. Figure 3D shows how the value of 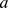 (the firing irregularity) changes as the balance between excitation and inhibition is varied.

**Figure 4:**
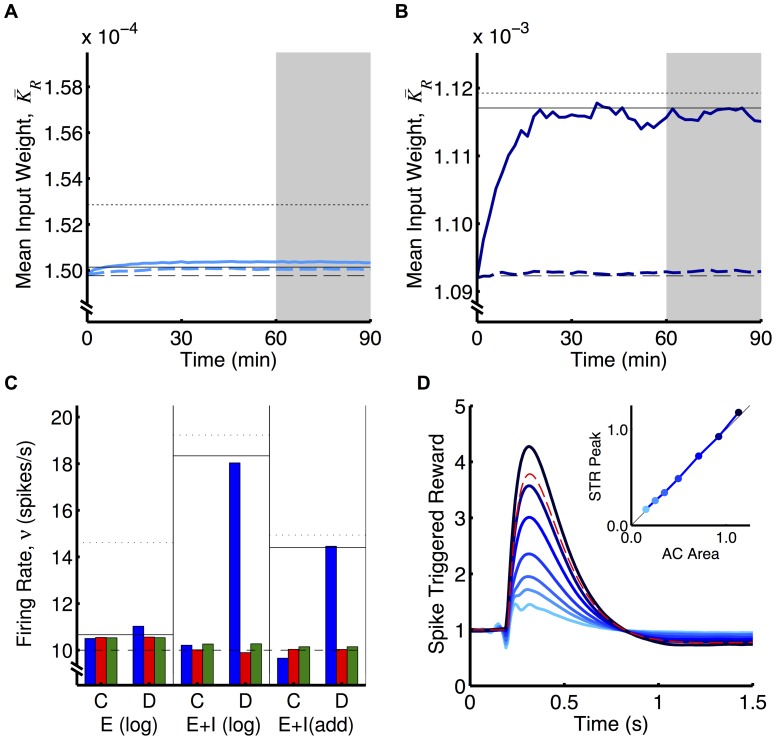
Operant conditioning experiment with LIF neurons. **A:** Mean weight into the reinforced neuron (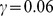) over time for LIF neurons receiving 10,000 excitatory inputs where the weights are updated using the classical (dashed) and dopamine (solid) RSTDP models. Horizontal lines represent analytical predictions for classical RSTDP (dashed), dopamine RSTDP where 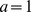 (dotted), and dopamine RSTDP where the correct value of 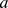 is assumed (solid). **B:** Same as A with 8,000 excitatory inputs and 2,000 inhibitory inputs (inhibitory synaptic strengths were fixed at 0.01). **C:** The mean firing rates of the reinforced (blue), surround (red), and control (green) neurons for the last 30 minutes of the simulations (shaded areas in A and B) with classical (C) and dopamine (D) RSTDP in A (E (log)), B (E+I (log)), and as in B but with additive weight dependence (E+I (add)), as described by Equation (19). Horizontal lines represent analytical predictions as in A and B. **D:** The average reward signal after the reinforced neuron's spikes (spike triggered reward) for neurons with different ratios between the excitatory and inhibitory input currents. The different ratios shown increase from no inhibitory inputs (lightest blue) up to the strongest inhibitory inputs (darkest blue), and correspond to the points in Figure 3D. The first line corresponds to the E case in A and C while the sixth line corresponds to the E+I case in B and C. The inset shows the relationship between the net area of the auto-covariance (AC) function and the peak of the spike triggered reward (STR) curve normalized by the peak of the reward kernel (red dashed line).

We compared the analytical predictions to simulations with LIF neurons (see Section 2 of Text S1 for derivation of the predicted weights/rates). While our analytical predictions hold for any weight dependence, for simulations we chose logLTD weight dependence (and also additive STDP). These results are shown in Figures 4A, 4B, and 4C. As predicted, classical RSTDP did not lead to an increase in the firing rate of the reinforced neuron in either E or E+I case. With dopamine RSTDP, this increase is seen but it is much smaller in the E case than in the E+I case. This has a number of causes, the most significant of which is that the negative region in the auto-covariance function, caused by the regular firing of the neuron in this case, almost completely cancels out the delta function at zero time lag (see Figure 3D), resulting in a small value for 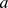. This has the effect of decorrelating the output spike train from itself and, therefore, the reward signal. This appears clearly in the average reward signal following spikes from the reinforced neuron (see Figure 4D). With low values of 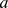 (regular firing), the inter-spike-intervals of the reinforced neuron are large and this causes the spikes to occur less during times of high reward. This is the reason that less reinforcement occurs in the E case.

Other reasons for the smaller amount of reinforcement observed in the E case (compared with the E+I case) are that the correlation strength decreases faster with mean weight and that a larger increase in the mean input weight is required for the same increase in the firing rate (Figures 3A and 3B). The latter of these influences is somewhat made up for by the larger value of 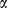 used in the E case. Figures 4A, 4B, and 4C include analytical predictions that assume 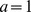 and others that take the correct value of 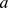 into account (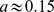 for E and 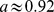 for E+I). This shows the contribution that the value of 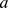, the irregularity of the firing, has on the reduced reinforcement in E compared with the other factors.

Cases E and E+I typify mean- and fluctuation-driven regimes, respectively, for the neurons. We observed that varying the relative amount of inhibitory input controls a smooth transition between these two regimes (Figure 3D). The correlation between the firing of the reinforced neuron and the reward signal and, therefore, the amount of reinforcement, perfectly follows this transition (Figure 4D).

Figure 4C also shows an example of this reinforcement learning with an additive weight dependence (E+I case only). This weight dependence includes rate-based learning terms, as used by Gilson et al. [20], and used slightly different modulation parameters to achieve stable equilibria (see Methods). These simulations show similar results as for the logLTD weight dependence.

### Correlated Inputs

**Figure 5:**
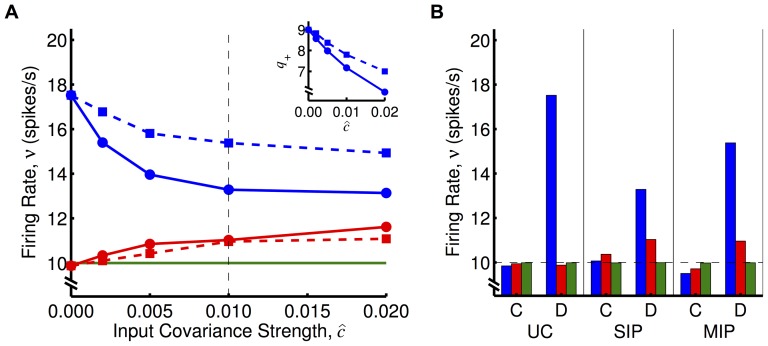
Operant conditioning experiment with correlations between inputs. **A:** Firing rates of reinforced (blue), surround (red), and control (green) neurons after learning in simulations with dopamine RSTDP for different input correlations (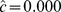, 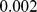, 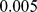, 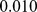, and 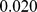) introduced using two different methods. The first method (SIP, solid with circles) leads to common spikes across many spike trains, while the second (MIP, dashed with squares) does not. *Inset*: Smaller values of the modulation offset, 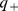, were used so that the stable firing rate of the control neurons remained at 10 spikes/s. **B:** Firing rates of the three neurons after learning with classical (C) and dopamine (D) RSTDP for uncorrelated inputs (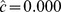) and with input correlation (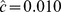, dashed vertical line in A) introduced using the two different methods.

We simulated the learning during the operant conditioning experiment where the inputs (excitatory and inhibitory) contained pairwise spike correlations and found that reinforcement still occurs and that the firing rate of the surround neuron also increased. We used two different methods of generating input correlations: the single and multiple interaction process (SIP and MIP, respectively) models [21]. Introducing correlations to the inputs leads to a higher firing rate even without providing the system with rewards. As shown in the inset of Figure 5A, we used smaller values of the modulation offset, 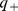, with dopamine RSTDP so that the stable firing rate of the control neurons remained at 10 spikes/s. For classical RSTDP, equal reductions were made to 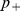 to achieve the same outcome. Figure 5A shows the resulting firing rates of the reinforced and surround neurons from simulations with different input correlations with dopamine RSTDP. Using either method, we observed a lower firing rate after learning for the reinforced neuron than for the uncorrelated case but reduction was larger with SIP correlations. We also observed an increase in the firing rate of the surround neurons above baseline (10 spikes/s) using either method. While this reduction may not have completely saturated with a covariance strength of 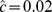, the trend appears to be sufficiently captured. Also, as the increase in the firing rate of surround neuron is due to its firing becoming correlated with the reinforced neuron's, our model does not predict that the surround neuron would ever increase its firing rate more than the reinforced neuron. Figure 5B shows the firing rates for only 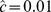 with both classical and dopamine RSTDP and compares them to the case with uncorrelated inputs. There is no apparent reinforcement of the firing rates of either neuron for classical RSTDP with input correlations.

### Non-Zero Reward Kernel Mass

**Figure 6:**
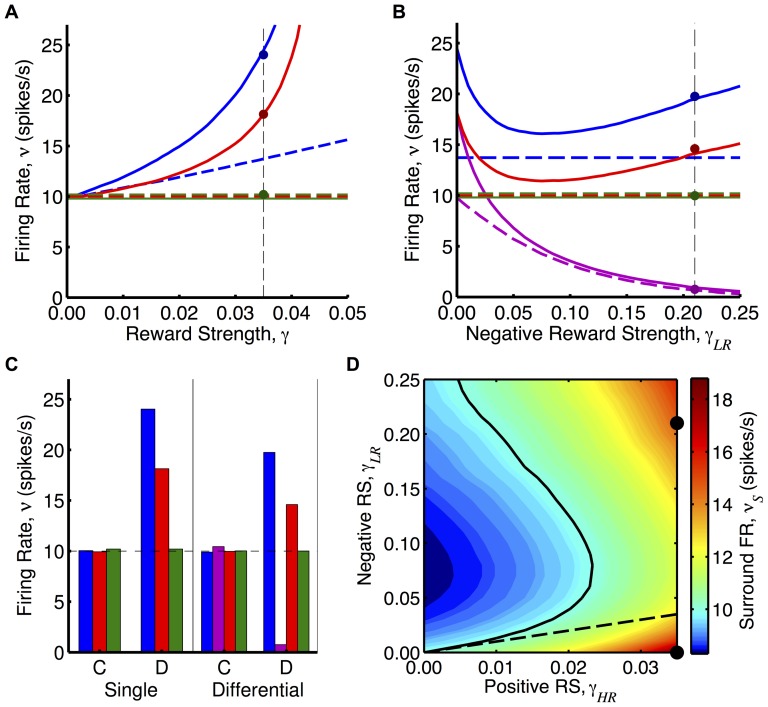
Operant conditioning experiment with non-zero-mass reward kernels. **A:** Firing rates of reinforced (blue), surround (red), and control (green) neurons after learning with dopamine RSTDP using reward kernels with masses of 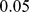 (solid) and 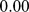 (dashed) with reward strength. The green solid line and the red and green dashed lines are shown separate but are actually in line. Dots show the firing rates after learning from a simulation using the 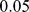 mass reward kernel. **B:** Same as A but with an additional neuron (magenta) that is reinforced for a low firing rate. The high-rate reinforced neuron has fixed reward strength of 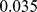, while the negative reward strength is varied. **C:** Firing rates of the three neurons (same colors as in A and B) after learning with classical (C) and dopamine (D) RSTDP for the single reinforced neuron, 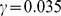, and differentially reinforced neurons, 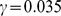 and 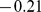 (vertical dashed lines and dots in A and B). **D:** Heat map of the firing rate (FR) of the surround neuron as the reward strengths (RSs) of the two neurons are varied. The solid line shows where the firing rate is unchanged from the base level (10 spikes/s) and the dashed line shows where the positive and negative reward strengths are equal in magnitude.

We found a similar result to adding correlated inputs, when we considered the case where the mass of the reward kernel, 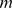, is no longer zero. In this case, the mean of the reward signal, 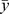, is not fixed at the base level, 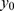. Instead, it is given by 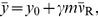
(4)


where 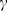 is the reward strength and 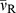 is the firing rate of the reinforced neuron. Figure 6A shows the analytical predictions for the mean firing rates of the neurons after learning for different reward strengths for 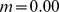 and 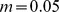. These results are supported by simulations, as shown in Figures 6A and 6C. For dopamine RSTDP, we observed that the firing rate of the surround neuron (as well as the reinforced neuron) increased above that of the control neuron when using a non-zero mass reward kernel. This was because the reward signal mean was no longer fixed but increased according to Equation (4). Because of this, we observed that the reinforced firing rate was unstable if the reward strength and kernel mass were too large. For classical RSTDP, neither the reinforced nor the surround firing rates increased.

### Differential Reinforcement

We also considered the case where there are two differentially reinforced neurons (i.e., the neurons have positive and negative reward strength, respectively). In this case, the mean reward is given by 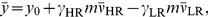
(5)


where 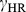 and 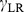 and 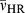 and 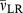 are the reward strengths and firing rates of the neurons reinforced for high and low firing rates, respectively. Figure 6B shows the analytical predictions for the mean firing rates of the four neurons (two differentially reinforced neurons and surround and control neurons) after learning for a positive reward strength of 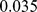 and different negative reward strengths for 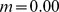 and 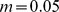. These results are supported by simulations, as shown in Figures 6B and 6C. As was the case with only one reinforced neuron, classical RSTDP did not lead to changes in the firing rates of any of the neurons. For dopamine RSTDP, we observed a decrease in the firing rate of the low-rate reinforced neuron, either for all values of 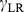 (with 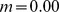) or for values of 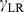 above a certain threshold (with 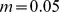), in addition to the increase in the firing rate of the high-rate reinforced neuron. Interestingly, as the negative reward signal increased, there was an initial decrease in the stable firing rate of the high-rate reinforced and surround neurons followed by a slow increase. This increase is due to the decreasing stable firing rate of the low-rate reinforced neuron having less of an effect on the mean of the reward signal. Figure 6D shows how the stable firing rate of the surround neuron depends on the two reward strengths. Depending on the two reward strengths, the stable firing rate of the surround neuron is above or below the firing rate of the control neuron.

### Comparison with R-max Model

As discussed by Fremaux et al. [7], the average change in synaptic weights due to reinforcement learning rules can be split into the unsupervised and reward learning components. The reward learning component depends on the covariance between neural activity and reward, while the unsupervised learning component is independent of this covariance, depending only the mean reward value. This separation of components is given by 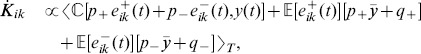
(6)


where 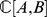 denotes the covariance between 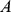 and 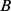, 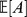 denotes the expected value of 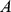 and 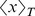 denotes the temporal average of signal 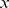. The first term in the equation is the reward learning component and the second and third terms combine to give the unsupervised learning component. For R-max and classical RSTDP, this simplifies to 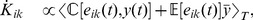
(7)


where 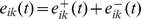. To maximize the reward that the system receives the unsupervised component needs to be as small as possible. The major difference between R-max and RSTDP is that, in the R-max model, the unsupervised component (or bias) is always zero (i.e., 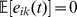). This is only possible because an assumption of the R-max model is that it has an unbiased estimator of the instantaneous firing rate of the post-synaptic neuron. In contrast, RSTDP is only able to have zero unsupervised bias if, in the classical case, the mean value of the reward signal is zero (or can be removed), or if, in our model, the mean value of the reward signal is such that 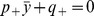 and 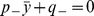. However, we are interested in when this is not the case and there is an unsupervised learning component. The unsupervised learning component without any reward learning leads to a stable base firing rate, and the introduction of the reward learning component, during operant conditioning, should result in a shift of this stable point. As we have shown, classical RSTDP is not able to both exhibit an ongoing unsupervised learning component that produces such a stable point and also elicit a shift in this stable point due to reinforcement learning.

**Figure 7:**
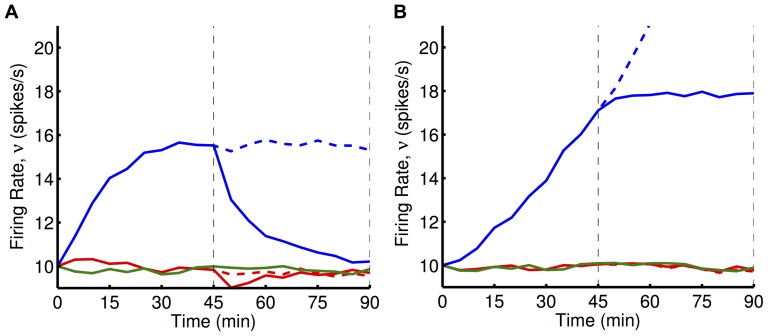
Comparison between dopamine RSTDP and R-max. **A:** Change in the firing rate over time for the reinforced (blue), surround (red), and control (green) neurons using the dopamine RSTDP model (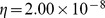), SRM neurons, and 10,000 excitatory inputs. A reward strength of 0.2 is used during the first 45 mins and this is either maintained for the second 45 mins (dashed) or reduced to 0.0 (solid). **B:** Same as A but using the R-max model (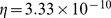).

In order to demonstrate how the operant conditioning experiment is different with and without an unsupervised learning component present, we used the Spike Response Model [22] to compare our dopamine RSTDP model (with logLTD) to the R-max model [7]. This is shown in Figure 7. Both models are able to elicit an increased firing rate in the reinforced neuron. For the same learning rate, the R-max model leads to much faster firing rate reinforcement so for comparison we have set the learning rate for the R-max model to be 60 times smaller than for the dopamine RSTDP model. Aside from the differences in learning rate and the size of the firing rate increase, there are two important differences between the models. They are both due to the fact that there is an unsupervised component (or bias) to the changes elicited by the dopamine RSTDP model but not with the R-max model. The first difference is that, using dopamine RSTDP, the firing rate returned to the base level during extinction, as observed in operant conditioning experiments [2]–[4], while in the R-max model it did not. The second difference is that the firing rate saturated in the dopamine RSTDP model, also as observed experimentally, while in the R-max model it did not. With our RSTDP model, there is a transient drop in the firing rate of the surround neuron at the beginning of the extinction period. This is due to a transient decrease in the mean value of the reward signal due to rewards no longer being delivered and the negative tail of the reward kernel. A transient increase in this firing rate similarly occurs at the beginning of the reinforcement period.

### Summary of Results

We considered RSTDP in the case where LTP and LTD exist, both without any rewards and also at the mean reward level, which means that unsupervised learning is present. We showed that, in this situation, classical RSTDP is not able to elicit the neuronal firing rate reinforcement that is observed in experiments and in models which assume that there is no unsupervised learning. We proposed a new RSTDP model, which better captures the experimentally observed modulation of STDP by dopamine, and showed that it is able to elicit firing rate reinforcement. Without any rewards, the unsupervised learning component led to a stable base firing rate (this was demonstrated with the control neuron) and, during an operant conditioning experiment, a reward learning component was introduced and, coexisting with the unsupervised learning component, led to a shift in the firing rate of the reinforced neuron. We identified that this reinforcement is much stronger when the neurons are in a fluctuation-driven regime (as opposed to a mean-driven regime), such as when they receive a balance of excitatory and inhibitory inputs. We demonstrated that our findings are robust to the weight dependency used, the input correlations, and whether the mean of the reward signal is fixed or dependent on the reinforced firing rate.

## Discussion

### Related Models of Operant Conditioning

Previous reinforcement learning models, such as classical RSTDP and R-max, are able to perform operant conditioning tasks only when they do not have an unsupervised component (or bias) to the synaptic changes they elicit [7], [16]. For R-max, this is the case regardless of the reward signal statistics, but, for classical RSTDP, this is only true when there is no LTP and LTD at the average reward value. However, there is much experimental evidence suggesting that unsupervised learning occurs in the brain. This includes all experiments in which STDP is observed to occur and especially the findings of Zhang et al. [19], which show that LTP and LTD are always present regardless of the dopamine concentration. An unsupervised learning component is also evident in the operant conditioning experiments when the reinforced firing rate returns to its original level during extinction [2], [3]. Figure 7 shows that our dopamine RSTDP model, with its unsupervised bias, can capture this behavior, unlike a model without an unsupervised component, such as R-max. A further aspect to the R-max model is that it requires an unbiased estimator of the instantaneous firing rate of the post-synaptic neuron in order to ensure there is never an unsupervised bias.

While a learning rule with an unsupervised learning component cannot always maximize the rewards received, it is not clear that learning rules employed by the brain are able to either. For example, in certain learning tasks, such as where perceptual roving is involved, R-max has been shown to out-perform the human brain [23]. This was our reason for considering the operant conditioning learning task in this paper. This simple situation can be compared directly with experiments and it is important to understand cases such as this before considering more general and complex learning situations. While out of the scope of this study, we would expect our model to perform similarly in more complex reinforcement learning tasks. As in this simple task, the unsupervised learning component would work against the reward-based changes but given sufficiently strong reinforcement learning components there is no reason why these learning tasks could not be performed.

Fremaux et al. argued that RSTDP is not an appropriate model of reward learning because it is sensitive to changes in the mean of the reward signal and will only work if the mean reward can be estimated without bias and subtracted from the current reward [7]. However, in the simple operant conditioning protocol corresponding to published experiments [2]–[4], we show that reward learning can coexist with unsupervised learning provided that certain conditions are imposed on how the STDP learning window changes with the value of the reward signal. Also, while Fremaux et al. considered a system in which rewards with positive mass (net area) were given and the mean reward over multiple trials had to be estimated and removed [7], we considered a model of dopamine dynamics in which this was unnecessary. Similar to Legenstein et al. [16], we assumed that rewards (bursts of dopamine) that the system received had zero mass, with dopamine dropping below baseline after an initial burst. This meant that the mean reward value was fixed and the presence of a critic to accurately estimate this mean (as discussed by Fremaux et al. [7]) was unnecessary.

### Reward Prediction

In the actual operant conditioning experiments, rewards are not given for each of the output spikes. However, visual feedback is presented to the monkey at the level of individual spikes and, through classical conditioning, we assume that the dopamine response comes to be elicited by the more frequent and earlier feedback of the spikes (conditioned stimuli) as this is predictive of the less frequent and delayed rewards (unconditioned stimuli). For this reason, we believe the reward signal we have used, in which kernels for each of the output spikes are summed, is consistent with the evidence that dopamine encodes reward prediction error (RPE) [6]. While dopamine ceases to be released for the actual rewards, no further predictor of the reinforced spikes exists and we expect that dopamine continues being released as these spikes occur.

We made the same type of assumptions for the case where a differential firing rate was being reinforced. As in the simple case, the visual feedback of the spikes is completely predictive of the rewards received. The only difference is that spikes from the neuron that is negatively punished for firing (the low-rate neuron) predict less (or later) rewards and so we assumed that these spikes should lead to a drop in the dopamine concentration.

### Firing Regimes

Neurons can operate as integrators that accumulate inputs over time to reach a threshold or as coincidence detectors that are sensitive to inputs arriving at the same time. These two different modes are referred to as mean-driven and fluctuation-driven regimes, respectively. In simple network models that only include excitatory synapses, neurons can only operate in a mean-driven regime, where firing is regular. However, when neurons receive a balance of excitatory and inhibitory inputs, they operate in a fluctuation-driven regime with high firing variability [24]–[26]. Experimental studies suggest that this is how cortical neurons operate [27], [28].

In this study, we found that firing rate reinforcement is stronger for irregular firing neurons. This is consistent with previous reinforcement learning studies [8], [9], [29], which found that firing variability is important for ensuring correlation between the reward signal and the neural firing to be reinforced. Here, we controlled the firing variability of LIF neurons by varying the relative amounts of excitatory and inhibitory inputs to the neurons.

In all the simulations in this study, the input firing rates (and the control firing rate) were 10 spikes/s. This was based on the observed firing rates in the corresponding experimental studies [2]–[4]. For lower firing rates, Equation (1) predicts a lower learning rate and a stronger influence of the cross-covariances between neurons and inputs, but it still predicts qualitatively similar outcomes for the firing rate changes.

### Experimental Predictions

We suggest three different types of possible experiments in which our model makes testable predictions. The first relates to the firing regime of the reinforced neuron. We predict that the effectiveness of the reinforcement learning is dependent on the firing regime of the neuron being reinforced. Fetz and Baker describe the reinforced neuron in their experiments as firing in bursts [3]. This type of firing regime would have an auto-covariance function with a net area greater than 1. This fits with our study, which predicts that this type of firing is beneficial to the reinforcement of firing rates (Figures 3D and 4D). To further test this prediction, operant conditioning experiments could be performed on neurons with different firing regimes, in particular, differently shaped auto-covariance functions. These could be different neurons, potentially in different brain regions, which are observed to naturally produce different firing behaviors. Alternatively, it may be possible to experimentally modify the firing statistics in a single neuron.

The second type of experiment relates to directly controlling a particular neuromodulator, such as dopamine, in the manner described in this paper and observing the firing rate changes. This would allow the RSTDP mechanism to be investigated more explicitly, without assuming the dopamine signal based on the reward scheme. As mentioned in the Introduction, other neuromodulators have been observed to affect STDP (see Figure 1A). It would be of particular interest to carry out this experiment with one of these modulators. This study predicts that neurons could either be reinforced or punished with the same reward signal depending on the neuromodulator and concentrations used. For example, a burst of octopamine could be injected into an area of the mushroom body of a locust for each spike from an arbitrarily chosen neuron such that it resembles the reward signal considered in this study. A similar experiment to this was performed by Nargeot et al., where an analogue of the operant conditioning of Aplysia was performed by stimulating the esophageal nerve, releasing dopamine [30].

The third type of experiment relates to the behavior of a nearby neuron, especially during the differential reinforcement experiment. During operant conditioning experiments, where a high firing rate was being reinforced, the firing rates of nearby neurons, which were not being reinforced, were also observed to significantly increase [3]. This increase was much more variable and in some cases was larger than the increase in the reinforced neuron. In our study, while the increase would never be more for the surround neuron than the reinforced neuron, this is consistent with there being correlated inputs (and, therefore, correlations between the neurons) or with a reward kernel with positive mass (and, therefore, an increase in the mean of the reward signal), or with both of these. Fetz and Baker qualitatively observed correlations between the neurons but did not carry out more quantitative measurements or analysis [3]. During the operant conditioning of the firing rate of a neuron, correlations between the reinforced neuron and a nearby neuron could be measured and compared with the increases of the firing rate of the two neurons. Alternatively, the firing of a nearby neuron could be controlled and made to fire independently of its inputs and, more importantly, independently of the reinforced neuron. After the firing rate of the reinforced neuron has increased, the control of the nearby neuron could be released and the firing rate that it exhibits immediately afterwards due to its inputs could be observed. Our model predicts that the firing rate of a nearby neuron will increase less if it is not correlated with the reinforced neuron. If there was still a firing rate increase, this would assumedly be due to an increase in the mean reward value. In this case, another experiment could be performed, observing the change in firing rate of a nearby neuron during the differential firing rate reinforcement of two neurons. Figure 6D shows that whether the firing rate of the surround neuron increased or decreased depended on the relative reward strengths of the two differentially reinforced neurons.

### Other Plasticity Models

We focussed on two specific weight dependencies (logLTD and additive), but Equation (1) holds for any pair of weight functions. Because the mechanism for the firing rate reinforcement is in the differential modulation of LTP and LTD, we would expect similar findings regardless of the weight dependence. It remains to be seen how more detailed models such as triplet STDP [31], [32] and voltage-based STDP [33] could be incorporated into RSTDP and how this would affect the results of this paper.

Building upon earlier models [34], [35], Graupner et al. proposed a synaptic plasticity model based on postsynaptic calcium concentrations of cells [36]. This biophysically based model is able to exhibit the results of many plasticity experiments relating to different STDP windows, pairing with postsynaptic spikes and bursts, triplet and quadruplet STDP, firing rate effects, and the effects of dendritic location. While our RSTDP model allows the change in the STDP learning window that Zhang et al. observed to occur with the addition of dopamine [19], this same dopamine dependence could be more simply incorporated by the modulation of just one of the parameters in the calcium-based plasticity model.

## Methods

### Neuron Models

We considered three neuron models: the Poisson neuron model, the leaky integrate-and-fire (LIF) neuron model, and the Spike Response Model (SRM) [22]. The Poisson neuron model was used in the analytical derivations, together with numerically determined functions for the firing rate and auto- and cross-correlations for the spike trains with mean input weight for the LIF neuron model. This aided the comparison between our analytical results and simulations with the LIF neuron model. The SRM is only used when comparing our RSTDP model to the R-max model.

The Poisson neuron model is a stochastic model that outputs a spike train that is a realization of an inhomogeneous Poisson process [37]. The intensity function of this process is analogous to the membrane potential of the neuron. It is made up of a spontaneous rate and the weighted sum of post-synaptic response kernels given by 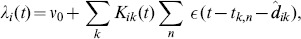
(8)


where 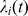 is the intensity function for the 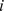
th neuron at time 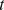, 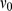 is the spontaneous rate (assumed to be zero in this study), 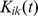 is the synaptic weight from input 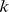 to neuron 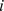, 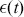 is the excitatory post-synaptic potential (EPSP) kernel, 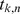 is the time of the 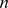
th spike output by neuron 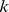, and 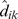 is the axonal delay from neuron 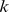 to neuron 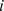. Synapses here are modeled as current based. This means that synaptic input into the neuron is independent of the neuron's membrane potential (the intensity function in this model). The EPSP kernel used in this study has the form 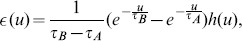
(9)


where 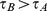 and 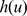 is the Heaviside function (i.e., 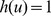 for 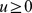 and 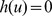 otherwise).

The leaky integrate-and-fire neuron is modeled using a single variable, 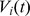. This represents the membrane potential for each neuron, 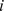, and evolves according to 
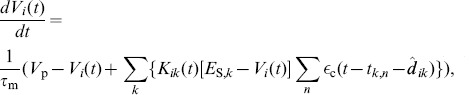
(10)


where 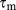 is the passive membrane time constant, 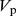 is the resting membrane potential, 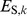 is the synaptic reversal potential of the (excitatory) synapses from neuron 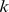, and 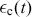 is the excitatory post-synaptic conductance (EPSC). The EPSC plays a similar role to the EPSP kernel, 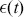, in the Poisson neuron model and, because of this, we refer to both 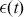 and 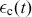 as EPSPs or EPSP kernels. 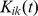, 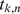, and 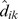 are the same as for the Poisson neuron model. A spike is produced when the membrane potential reaches a threshold value, 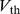, and it is reset to 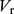. An absolute refractory period is used, which prevents the membrane potential from changing during this time. The values of these parameters are given in Table 1. Similarly, the parameters for the Spike Response Model (the same as those used by Fremaux et al. [7]) are shown in Table 2. Simulations with the LIF neuron model and the SRM were performed using an in-house neuron modeling software program, SpikeSim, used in previous studies [20], [38]–[40].

**Table 1:** LIF Neuron Parameters.

Parameter	Value
Synaptic Rise and Decay Times: 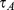 , 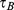 (ms)	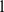 , 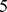
Membrane Time Constant: 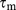 (ms)	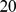
Threshold, Resting and Reset Potentials: 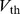 , 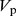 , 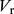 (mV)	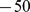 , 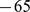 , 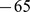
Excitatory/Inhibitory Reversal Potentials: 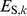 (mV)	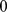 , 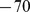
Refractory Period (ms)	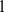

**Table 2:** SRM Neuron Parameters.

Parameter	Value
Synaptic Rise Time: 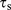 (ms)	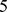
Membrane Time Constant: 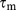 (ms)	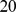
Firing Rate at Threshold: 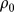 (spikes/s)	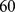
Threshold and Reset Potentials: 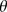 , 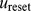 (mV)	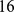 , 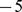
Escape Noise Control: 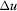 (mV)	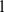

We considered the feed-forward network shown in Figure 1E, which has three different post-synaptic neurons: the reinforced, surround, and control neurons. Unless otherwise stated, we have considered the case where there is a single reinforced neuron and an arbitrary number of surround and control neurons (the number does not affect the results). Each neuron outputs a spike train, 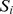, with a mean firing rate, 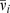. They receive synaptic inputs from 10,000 input spike trains, 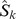, with strength, 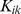, and equal axonal delay, 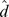 (dendritic delays are assumed to be negligible). The input spike trains are assumed to be uncorrelated and have the same mean firing rate, 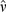. The mean feed-forward weights and mean firing rates of the reinforced, surround, and control neurons are denoted 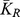 and 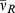, 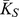 and 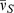, and 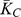 and 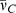, respectively. In simulations, the weights are initially the same and set to be approximately equal to 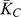.

### Reward Signal

As in previous studies [16], we assumed that rewards given to the monkey affect the concentration of dopamine in the neural network. This is based upon the evidence linking dopamine to reward learning in the brain [6]. Dopamine is delivered to different brain regions by the axons of neurons located in the ventral tegmental area (VTA), whose activity is dependent not only on rewards received but also on predicted or expected rewards.

In the operant experiments by Fetz and Baker, and Kobayashi et al. [2]–[4], monkeys were presented with a screen showing a signal that decayed with time but was incremented for each action potential measured from an electrode implanted in their precentral motor cortex or prefrontal cortex. If the signal reached a threshold value, a reward was given and the signal returned to a reset value. With this setup, the experiments showed that high firing rates were elicited. Negative punishment (i.e., the removal of reward in order to decrease a particular behavior) was performed with a similar setup, where measured spikes decremented the signal (and artificially generated spikes incremented the signal). In this case, low firing rates were elicited. Through a combination of positive reinforcement and negative punishment, they also showed that a differential between the firing rates of two neurons could be elicited.

In our model, the reward signal, which is related to the dopamine concentration, is driven by the firing of the reinforced neuron(s) and is given by 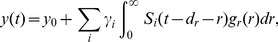
(11)


where 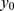 is the base level of the reward signal, 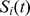 is the spike train of reinforced neuron 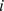, 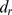 is the reward delay, and 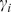 is the reward strength for neuron 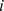 (this can be either positive or negative for neurons whose firing affects the signal, or zero for neurons whose firing does not). Reward strengths correspond to the heights of the voltage pulses delivered to the feedback signal for each spike of reinforced neurons in the operant conditioning experiments [2], [3]. The reward kernel, 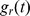, is given by 
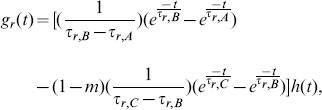
(12)


where 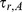, 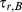, and 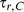 are the rise, decay, and recovery time constants, respectively, and 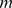 is the normalized kernel mass. As in the study by Legenstein et al. [16], we initially focussed on the case where the reward kernel has zero mass (i.e., 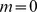 and so 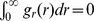). If this is the case, the mean of the dopamine signal is fixed (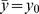). This dopamine signal affects the synapses to the reinforced and surround neurons but not the control neurons. The dopamine signal that affects the control neuron(s) is one that remains fixed at the base level, 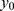. The reward kernel parameters used in this study are shown in Table 3. Figure 1D shows an example of a reward signal, 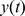, dependent on the spike train of neuron 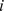, 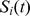.

**Table 3:** RSTDP Parameters.

Parameter	Value
Reward Rise, Decay & Recovery Times: 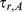 , 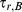 , 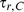 (s)	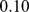 , 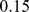 , 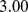
Reward Delay: 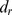 (s)	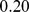
Base Reward Level: 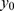	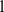
Eligibility Rise and Decay Times: 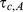 , 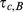 (s)	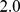 , 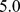
LTP/LTD Window Time Constants: 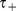 , 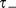 (ms)	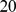 , 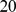
LogLTD Parameters (E): 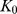 , 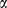	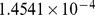 , 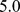
LogLTD Parameters (E+I): 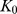 , 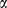	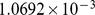 , 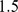
LogLTD Parameters (SRM): 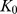 , 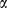	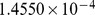 , 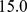
Additive Input/Output Rate Parameters: 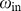 , 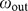	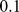 , 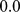
Dopamine Modulation Parameters (log): 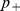 , 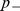 , 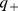 , 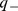	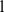 , 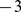 , 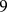 , 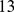
Classical Modulation Parameters (log): 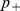 , 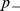 , 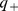 , 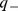	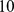 , 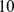 , 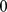 , 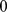
Dopamine Modulation Parameters (add): 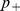 , 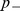 , 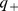 , 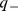	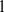 , 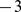 , 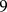 , 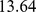
Classical Modulation Parameters (add): 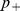 , 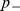 , 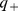 , 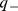	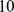 , 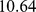 , 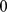 , 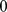

### RSTDP Model

Based upon the experimental results of Zhang et al. [19], Figure 1B shows the observed effect that the concentration of dopamine has on the amplitudes of LTP and LTD (blue circles). These experimental observations suggest that LTD and LTP are non-zero when there is no dopamine, that as the concentration of dopamine increases, LTD and LTP change in different ways, and that for high dopamine concentrations, LTD switches to LTP. In addition to dopamine, other neuromodulators have been observed to affect STDP. These neuromodulators include acetycholine [41] in the hippocampus of rats, octopamine in the mushroom body of locusts [42], and norepinephrine in the visual cortex of mice [43]. Their effects on LTP and LTD are illustrated with the markers in Figure 1A.

In the existing RSTDP model, “classical RSTDP”, both LTP and LTD are modulated equally by the reward signal (i.e., the dopamine concentration) such that no synaptic changes can occur when there is no reward. This is illustrated in Figure 1A (dashed blue line). Figure 1C shows this as different learning windows (relationships between the timing difference of spike pairs and the change in synaptic weight) for different dopamine concentrations. This paper introduces a new RSTDP model that can better capture experimental findings [19], [41]–[43]. In our RSTDP model, the potentiation (LTP) and depression (LTD) parts of the STDP learning window (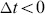 and 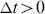, respectively) are modulated separately by the reward signal. This new model is shown in Figure 1A (solid blue line) and with different learning windows in Figure 1B.

In our RSTDP model, changes to the feed-forward weights are given by 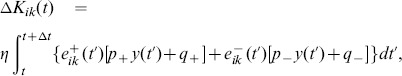
(13)


and so the time and ensemble averaged rate of change of these feed-forward weights is given by 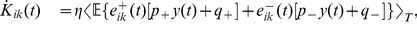
(14)


where 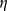 is the learning rate, 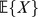 is the expected value of a random variable 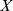, and 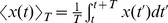 is the temporal average of the signal, 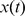, over a timescale, 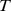, that is slower than both the neuronal and reward signal dynamics. The eligibility traces for LTP and LTD are given by 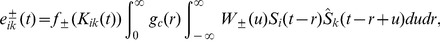
(15)


where 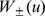 and 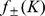 are the learning windows and weight dependence functions for LTP (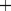) and LTD (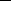), respectively. The modulation offsets, 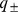, give the amplitude of LTP and LTD for zero reward, while the modulation slopes, 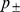, describe how the reward signal affects the amplitudes of LTP and LTD, respectively. The eligibility kernel, 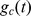, is given by 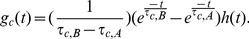
(16)


This learning process is described in Figure 1D.

The learning window, which is divided into the LTP and LTD windows, is given by 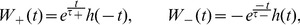
(17)


where 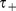 and 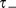 are the time constants for LTP and LTD, respectively. As the relative amplitudes of LTP and LTD are determined by the modulation parameters, the amplitudes of the learning windows were both set to to 1 to avoid redundancy in the parameters. For the same reason, the base value of the reward signal (which for zero-mass reward kernels is equal to the signal mean) is set to 1.

The type of weight dependence, 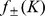, that we focussed on in this paper was one with additive LTP and logarithmically dependent LTD. This was inspired by the weight dependence considered by Gilson et al. [44]. This weight dependence is referred to as “logLTD”. The functions for logLTD are given by 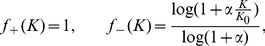
(18)


where 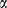 and 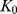 are parameters defining the shape of the LTD weight dependence. This weight dependence was chosen because it provides an intermediate between additive and multiplicative weight dependencies. Additive STDP leads to strong competition between the synapses and a bimodal weight distribution. Multiplicative STDP leads to a unimodal weight distribution but only weak competition [44]. LogLTD elicits strong competition between the synapses, while producing a stable, unimodal weight distribution. We also considered additive weight dependence, where the functions are given by 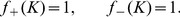
(19)


Additive weight dependence was considered with rate-based learning terms [37], which are not modulated by the reward signal. These are given by 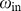 and 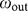, which either increase or decrease the synaptic weight for each pre- or post-synaptic spike, respectively. When using an additive weight dependence, these rate-based terms are necessary to achieve a stable mean weight.

The parameters values for the eligibility kernel, learning window, and weight dependence functions are shown in Table 3 (the parameters of the weight dependence functions were chosen to produce the desired stable firing rate for the control neuron and to exhibit sufficient sensitivity to being reinforced). Equation (1) was derived from Equations (11), (14) and (15) using results from Bohrnstedt and Goldberger [45] (see Section 1 of Text S1). The analytical predictions for the resulting mean input weights, for the two different weight dependencies in Equations (18) and (19), are based on Equation (1) (see Section 2 of Text S1).

### Covariances in the Network

We have focussed on the case where the inputs are uncorrelated and the neurons receive separate (non-overlapping) sets of input spike trains. While the inputs are uncorrelated, correlations between the neurons and inputs arise due to the influence of individual input spikes on the firing of the output neuron. These are referred to as “spike triggered correlations”. Therefore, for neurons 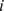 and 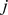 and one of the inputs, 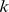, into neuron 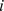, we have mean neuron-input cross-covariances, 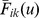 and 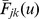, and mean neuron-neuron auto- and cross-covariances, 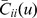 and 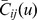, given by 
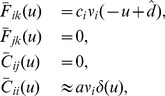
(20)


where 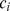 is the magnitude of the spike triggering effect, 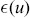 is the EPSP kernel, and 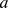 is net area of the auto-covariance function of neuron 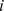 for short time lags. For Poisson neurons, 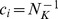, where 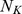 is the number of input spike trains into each neuron. However, for LIF neurons, 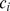 is not constant but depends on the strength of the inputs into neuron 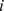. Figures 3A and 3B show numerically determined values for 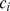 when there are only excitatory inputs and when there is a balance of excitatory and inhibitory inputs, respectively. For Poisson neurons, 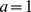, while for LIF neurons, this is not necessarily the case. This discrepancy is often due to the minimum inter-spike interval that LIF neurons exhibit. While we approximated 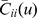 as a delta function, Figure 3C shows that this is not the case on short time scales. Figure 3D shows how 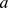 and the shape of the auto-covariance function change with the ratio of inhibitory to excitatory input currents. These curves agree with analytical studies that considered the statistics of LIF neuron outputs [46], [47].

For correlated inputs, 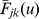 and 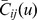 would no longer be zero and new curves for the output firing rate and the neuron-input and neuron-neuron covariance strengths with mean input weight would need to be determined. While this would be more complex, the analytical framework presented is able to incorporate these differences and make predictions for reinforcement learning with input correlations. However, in this study, we considered operant conditioning experiments with correlated inputs through simulations only, and did not analytically derive expressions for this case. In these simulations, we considered two methods for generating inputs with constant firing rates and pairwise covariances. The first, referred to as the single interaction process (SIP) model, introduces the pairwise covariances between inputs through common spike events, in which many inputs participate [21], [48], [49]. The second, referred to as the multiple interaction process (MIP) model, introduces pairwise covariances without these common spike events [21]. We considered input correlations of up to 0.02, consistent with the range of correlations typically observed in the cortex [50].

## Supporting Information

Text S1 **Analytical derivations.** (PDF). Sections: (1) Derivation of Learning Equation, and (2) Resulting Mean Input Weights.
(PDF)
