# Supplementary material for: Coexistence of Reward and Unsupervised Learning During the Operant Conditioning of Neural Firing Rates
Source: PLoS One. 2014 Jan 27;9(1):e87123. doi: 10.1371/journal.pone.0087123 (PMC3903641; doi:10.1371/journal.pone.0087123)
Supplement: Text S1 — Analytical derivations. (PDF). Sections: (1) Derivation of Learning Equation, and (2) Resulting Mean Input Weights. (PDF) [file pone.0087123.s001.pdf]

Supporting Information: Text S1

1 Derivation of the Learning Equation

Given the general RSTDP model (Equations (14) and (15) of the main text), a description of how the reward signal depends on the network activity (Equation (11) of the main text), and the details of the neuron models, we derived the specific learning equations that govern how a network evolves in time. We considered only the case where both the firing rates and the correlations between neurons are quasi-stationary (change very slowly over time) and are negligible for large time lags (of the order of the reward delay, d_r). Different definitions for the learning window, and correlations and covariances were used by Legenstein et al. and Gilson et al. [1,2]; the time lags of the functions are reversed. We used the definitions from the latter.

We first considered the learning due to an arbitrary reward signal with fixed mean. This is given by

$$\dot{K}_{ik} = \eta \bigg\{ \int_0^\infty g_c(s) \Big[p_+ f_+(K_{ik}) Y_{ik}^{W_+}(t,s) + p_- f_-(K_{ik}) Y_{ik}^{W_-}(t,s) \Big] ds \\ + (p_+ \bar{y} + q_+) f_+(K_{ik}) D_{ik}^{W_+}(t) + (p_- \bar{y} + q_-) f_-(K_{ik}) D_{ik}^{W_-}(t) \bigg\}, \tag{1}$$

where $\hat{d}$ is the axonal delay from the inputs, $D_{ik}^{\psi}(t) = \int_{-\infty}^{\infty} \psi(u) D_{ik}(t, u - \hat{d}) du$, $D_{ik}(t,u)$ is the neuron-input cross-correlation (i.e., $D_{ik}(t,u) = F_{ik}(u) + \nu_i \hat{\nu}_k$), $Y_{ik}^{\psi}(t,s) = \int_{-\infty}^{\infty} \psi(u) Y_{ik}(t,s,u-\hat{d}) du$, and $Y_{ik}(t,s,u)$ is given by

$$Y_{ik}(t,s,u) = \Big\langle \mathbb{E}\big[\Delta y(t) S_i(t-s) \hat{S}_k(t-s+u)\big] \Big\rangle_T, \tag{2}$$

where $\Delta y(t) = y(t) - \bar{y}$.

For the operant conditioning experiment, we substituted Equation (11) of the main text into Equation (2) to give

$$Y_{ik}(t,s,u) = \sum_j \gamma_j \int_0^\infty g_r(r) \mathbb{E}\Big[S_j(t - d_r - r) S_i(t-s) \hat{S}_k(t-s+u) \Big] dr. \tag{3}$$

Using the results of Bohrnstedt and Goldberger [3], we found that

$$\mathbb{E}\big[S_j(t_1) S_i(t_2) \hat{S}_k(t_3)\big] = \mathbb{E}\big[S_j(t_1)\big] \mathbb{E}\big[S_i(t_2)\big] \mathbb{E}\big[\hat{S}_k(t_3)\big] + \mathbb{E}\big[S_j(t_1)\big] \mathbb{C}\big[S_i(t_2), \hat{S}_k(t_3)\big] \\ + \mathbb{E}\big[S_i(t_2)\big] \mathbb{C}\big[S_j(t_1), \hat{S}_k(t_3)\big] + \mathbb{E}\Big[\hat{S}_k(t_3)\Big] \mathbb{C}\big[S_j(t_1), S_i(t_2)\big] \\ + \mathbb{C}\big[S_j(t_1), S_i(t_2), \hat{S}_k(t_3)\big], \tag{4}$$

where $\mathbb{C}[A,B]$ is the joint cumulant of random variables A and B (the covariance), and $\mathbb{C}[A,B,C]$ is the joint cumulant of random variables A, B, and C.

We have assumed that only pairwise correlations exist (i.e. for $i \neq j$, $\mathbb{C}\big[S_j(t_1), S_i(t_2), \hat{S}_k(t_3)\big] = 0$). However, when $i = j$, we found that

$$\mathbb{C}\big[S_i(t_1), S_i(t_2), \hat{S}_k(t_3)\big] = \nu_i^{-1} \bar{C}_{ii}(t_2 - t_1) \bar{F}_{ik}(t_3 - t_1). \tag{5}$$

Therefore, using the covariances given in Equation (20) of the main text, we derived that

$$\begin{aligned}\mathbb{E}\big[S_i(t_1)S_i(t_2)\hat{S}_k(t_3)\big] &= \nu_i\nu_i\hat{\nu} + \nu_i\bar{F}_{ik}(t_3-t_2) + \nu_i\bar{F}_{ik}(t_3-t_1)\\ &\quad + \hat{\nu}\bar{C}_{ii}(t_2-t_1) + \nu_i^{-1}\bar{C}_{ii}(t_2-t_1)\bar{F}_{ik}(t_3-t_1)\\ &= \nu_i\nu_i\hat{\nu} + c_i\nu_i\nu_i\epsilon(t_2-t_3+\hat{d}) + c_i\nu_i\nu_i\epsilon(t_1-t_3+\hat{d})\\ &\quad + a\hat{\nu}\nu_i\delta(t_2-t_1) + ac_i\nu_i\delta(t_2-t_1)\epsilon(t_1-t_3+\hat{d}),\end{aligned} \tag{6}$$

and

$$\begin{aligned}\mathbb{E}\big[S_j(t_1)S_i(t_2)\hat{S}_k(t_3)\big] &= \nu_j\nu_i\hat{\nu} + \nu_j\bar{F}_{ik}(t_3-t_2) + \nu_i\bar{F}_{jk}(t_3-t_1) + \hat{\nu}\bar{C}_{ij}(t_2-t_1)\\ &= \nu_j\nu_i\hat{\nu} + c_i\nu_j\nu_i\epsilon(t_2-t_3+\hat{d}).\end{aligned} \tag{7}$$

Substituting Equations (6) and (7) into Equation (3), we derived an expression for $Y_{ik}(t,s,u)$ as

$$Y_{ik}(t,s,u) = \gamma_i\nu_i\Big\{ag_r(s-d_r)\big[\hat{\nu} + c_i\epsilon(-u+\hat{d})\big] + c_i\nu_i g_r(s-d_r-u+\hat{d})\Big\}, \tag{8}$$

where $\epsilon(t)$ is the excitatory post-synaptic potential (EPSP) and c_i is the mean correlation strength between neuron i and its inputs. This also includes a correction factor, $a = \int_{-\mathcal{U}}^{\mathcal{U}} \bar{C}_{ii}(t,u)du$, where $\bar{C}_{ii}(t,u)$ is the mean auto-covariance function of neuron i at time t, and $\mathcal{U}$ is a period of time longer than the time scale of the learning window but shorter than the time scale of the reward and eligibility kernels. For Poisson neurons with constant inputs, the auto-covariance is a simple delta-function and so $a = 1$. However, for LIF neurons, this is not necessarily the case.

We substituted Equation (8) into Equation (1) and obtained the rates of change of the mean weights into the reinforced, surround, and control neurons as, respectively,

$$\begin{aligned}\dot{\bar{K}}_R &= \eta\bar{\nu}_R\Big\{\big[p_+(\bar{y}+a\gamma\eta_r)+q_+\big]f_+(\bar{K}_R)\big[\tilde{W}_+\hat{\nu}+\bar{c}_R\theta\big] + \big[p_-(\bar{y}+a\gamma\eta_r)+q_-\big]f_-(\bar{K}_R)\tilde{W}_-\hat{\nu}\\ &\qquad + \gamma\eta_r\bar{\nu}_R\bar{c}_R\big[p_+f_+(\bar{K}_R)\tilde{W}_+ + p_-f_-(\bar{K}_R)\tilde{W}_-\big]\Big\},\\ \dot{\bar{K}}_S &= \eta\bar{\nu}_S\Big\{\big[p_+\bar{y}+q_+\big]f_+(\bar{K}_S)\big[\tilde{W}_+\hat{\nu}+\bar{c}_S\theta\big] + \big[p_-\bar{y}+q_-\big]f_-(\bar{K}_S)\tilde{W}_-\hat{\nu}\Big\},\\ \dot{\bar{K}}_C &= \eta\bar{\nu}_C\Big\{\big[p_+y_0+q_+\big]f_+(\bar{K}_C)\big[\tilde{W}_+\hat{\nu}+\bar{c}_C\theta\big] + \big[p_-y_0+q_-\big]f_-(\bar{K}_C)\tilde{W}_-\hat{\nu}\Big\},\end{aligned} \tag{9}$$

where $\bar{y}$ is the mean value of the reward signal, $\tilde{W}_+$ and $\tilde{W}_-$ are the integrals over the LTP and LTD parts of the learning window, respectively, $\eta_r = \int_0^\infty g_c(s)g_r(s-d_r)ds$, $\theta = (W_+ * \epsilon)(0)$, and $\bar{\nu}_R$ and $\bar{c}_R$, $\bar{\nu}_S$ and $\bar{c}_S$, and $\bar{\nu}_C$ and $\bar{c}_C$ are the mean firing rates and mean spike triggered correlations of the reinforced, surround, and control neurons, respectively. For the specific functions and kernels used in this study, $\eta_r = 0.76$ and $\theta = 0.76$. For the small covariances due to the spike triggering effect, the third term for the evolution of $\bar{K}_R$ in Equation (9) can be neglected and this gives Equation (1) of the main text.

2 Resulting Mean Input Weights

Using logLTD weight dependence (Equation (18) of the main text) and uncorrelated input spike trains, the equations describing the stable equilibria of the mean synaptic weights into the reinforced, surround, and control neurons, $\bar{K}_R$, $\bar{K}_S$, and $\bar{K}_C$, respectively, are

$$\begin{aligned}
\frac{\log(1+\alpha\bar{K}_R^*/K_0)}{\log(1+\alpha)} &= \frac{\gamma\eta_r\bar{\nu}_R^*\bar{c}_R^*p_+\tilde{W}_+ + \left[p_+(\bar{y}+a\gamma\eta_r)+q_+\right]\left[\tilde{W}_+\hat{\nu}+\bar{c}_R^*\theta\right]}{\gamma\eta_r\bar{\nu}_R^*\bar{c}_R^*p_-\tilde{W}_- + \left[p_-(\bar{y}+a\gamma\eta_r)+q_-\right]\tilde{W}_-\hat{\nu}}, \\
\frac{\log(1+\alpha\bar{K}_S^*/K_0)}{\log(1+\alpha)} &= \frac{\left[p_+\bar{y}+q_+\right]\left[\tilde{W}_+\hat{\nu}+\bar{c}_S^*\theta\right]}{\left[p_-\bar{y}+q_-\right]\tilde{W}_-\hat{\nu}}, \\
\frac{\log(1+\alpha\bar{K}_C^*/K_0)}{\log(1+\alpha)} &= \frac{\left[p_+y_0+q_+\right]\left[\tilde{W}_+\hat{\nu}+\bar{c}_C^*\theta\right]}{\left[p_-y_0+q_-\right]\tilde{W}_-\hat{\nu}},
\end{aligned} \tag{10}$$

where K_0 and α are the parameters of the weight dependence function.

Using additive weight dependence (Equation (19) of the main text) with rate-based learning terms, we have the equilibria

$$\begin{aligned}
\frac{\bar{\nu}_R^*}{\hat{\nu}} &= \frac{-\omega_{\rm in}}{\left[p_+(\bar{y}+a\gamma\eta_r)+q_+\right]\left[\tilde{W}_+\hat{\nu}+\bar{c}_R\theta\right] + \left[p_-(\bar{y}+a\gamma\eta_r)+q_-\right]\hat{\nu}\tilde{W}_- + \omega_{\rm out}}, \\
\frac{\bar{\nu}_S^*}{\hat{\nu}} &= \frac{-\omega_{\rm in}}{[p_+\bar{y}+q_+][\hat{\nu}\tilde{W}_+ + \bar{c}_S\theta] + [p_-\bar{y}+q_-]\hat{\nu}\tilde{W}_- + \omega_{\rm out}}, \\
\frac{\bar{\nu}_C^*}{\hat{\nu}} &= \frac{-\omega_{\rm in}}{[p_+y_0+q_+][\hat{\nu}\tilde{W}_+ + \bar{c}_C\theta] + [p_-y_0+q_-]\hat{\nu}\tilde{W}_- + \omega_{\rm out}},
\end{aligned} \tag{11}$$

where the rate-based learning terms, $\omega_{\rm in}$ and $\omega_{\rm out}$, give the changes to the synaptic strength for pre- and post-synaptic spikes, respectively.

References

1. Legenstein R, Pecevski D, Maass W (2008) A learning theory for reward-modulated spike-timing-dependent plasticity with application to biofeedback. PLoS Comput Biol 4: e1000180.

2. Gilson M, Burkitt AN, Grayden DB, Thomas DA, van Hemmen JL (2009) Emergence of network structure due to spike-timing-dependent plasticity in recurrent neuronal networks II: Input selectivity–symmetry breaking. Biol Cybern 101: 103–114.

3. Bohrnstedt GW, Goldberger AS (1969) On the exact covariance of products of random variables. J Am Stat Assoc 64: 1439–1442.
